# Analysis of endogenous hormones and transcriptomes involved in *in vitro* shoot apical dormancy during adventitious root formation in tree peony

**DOI:** 10.3389/fpls.2025.1610747

**Published:** 2025-09-11

**Authors:** Yujie Yang, Yanan Gu, Ping Gao, Lan Chen, Qingxuan Zhang, Jie Luo, Dapeng Miao, Shusheng Wen, Yu Duan

**Affiliations:** ^1^ College of Landscape Architecture, Nanjing Forestry University, Nanjing, China; ^2^ Tianyi Ecological Science and Technology Institute, Sichuan Tianyi Ecological Garden Group Co., Ltd., Chengdu, China; ^3^ Landscape Architecture Institute, Chengdu Municipal Engineering Design & Research Institute Co., Ltd., Chengdu, China; ^4^ Chengdu Architectural Design & Research Institute Co., Ltd., Chengdu, China

**Keywords:** tree peony, shoot apical dormancy, root induction, hormone, transcriptome analysis

## Abstract

Tree peony (*Paeonia* sect. *Moutan*) is widely cultivated worldwide. However, conventional propagation methods such as sowing, division, and grafting are constrained by low reproduction rates and long reproductive cycles. Thus, micropropagation technology is a viable alternative to advance the tree peony industry. However, the industrial production of tree peony through this technique is largely limited by shoot apical dormancy in *in vitro* plantlets, and its molecular mechanism remains unclear. In this study, changes in endogenous hormone content during adventitious root formation were investigated in *Paeonia × lemoinei* ‘High Noon’. Transcriptome sequencing was carried out at four stages of root induction (0 d, 10 d, 20 d, 30 d) using Illumina HiSeq. The results showed that a decrease in trans-zeatin riboside (ZR) and gibberellic acid (GA_3_) content induced shoot apical dormancy. In contrast to previous studies, high levels of abscisic acid (ABA) were not the dominant factor inducing dormancy in *in vitro* tree peony plantlets. The accumulation of indole-3-acetic acid (IAA) in shoot apices promoted dormancy by activating the ABA signaling pathway without enhancing ABA levels. A total of 92.07 Gb of clean data were obtained, and 121,843 unigenes were assembled. The regulation of shoot apical dormancy is governed by core metabolic pathways, including plant hormone signal transduction, starch and sucrose metabolism, and phenylpropanoid biosynthesis. In these pathways, 27, 18, and 17 differentially expressed genes (DEGs) were identified, respectively. Based on the endogenous hormone content in the apical shoot and RNA-seq data collected during shoot apical dormancy, a preliminary model was constructed to illustrate how endogenous hormones regulate *in vitro* shoot apical dormancy in tree peony. These results provide rich gene resources for investigating the molecular mechanism underlying *in vitro* plantlet dormancy and will significantly contribute to advancing the tree peony industry by improving the transplant survival rate of micropropagation.

## 1 Introduction

Tree peony (*Paeonia* sect. *Moutan*) is a precious woody plant renowned for its ornamental value, medicinal use, and edible oil production. Native to China, it is now well-received by consumers worldwide. Currently, traditional propagation methods for tree peonies—such as seeding, division, and grafting—are inefficient and unable to meet the increasing market demand. Micropropagation serves as an efficient tool for the rapid and large-scale multiplication of plants and has been extensively adopted to address the shortcomings of traditional propagation methods. Nevertheless, a low survival rate has hindered the industrial application of this technology in tree peony, largely due to *in vitro* shoot apical dormancy induced during root induction (Wen et al., 2020).

In tree peony, *in vitro* shoot apical dormancy was first reported by Bouza et al. (1992), who found that the mitotic index in shoot apices significantly reduced during root induction, accompanied by endogenous abscisic acid (ABA). Without breaking dormancy, rooted shoots failed to grow and gradually died after transplanting. Since then, several studies have addressed shoot apical dormancy (Bouza et al., 1992). Thereafter, copious research was reported about the apical dormancy cultivation (Beruto et al., 2004; Wang et al., 2016; Wen et al., 2016a, Wen et al., 2018; Xin et al., 2019). Currently, many studies have attempted to break the *in vitro* shoot apical dormancy of tree peony by chilling treatment. It was found that although some growth could be achieved in plantlets due to raised mitotic index and reduced ABA production, after two months of *ex vitro* establishment, they would again enter dormancy and eventually die (Bouza et al., 1992, Bouza et al., 1994; Wen et al., 2016b; Wang et al., 2016). In conclusion, the *in vitro* shoot apical dormancy remains a major bottleneck in the industrial application of tree peony micropropagation, and chilling treatments have proven ineffective. Given the limitations of previous cytological and physiological studies in elucidating the underlying mechanisms, there is an urgent need to investigate the regulatory pathways of shoot apical dormancy from a molecular perspective. The molecular study started fairly late in the dormancy mechanism of tree peony. The current research mainly focus on the activation of the gibberellin signal transduction pathway and the mechanisms of bud dormancy release. These include the molecular cloning and functional prediction of genes such as *PsGA20ox* (Appleford et al., 2006), *PsSOC1* (Wang et al., 2015), *PsSERK2* (Gao et al., 2018), and *PsGAI* (Chi et al., 2021). Other efforts have characterized microRNAs involved in chilling-induced dormancy release (Zhang et al., 2018a), conducted transcriptome analyses, and screened relevant ERF transcription factors (Zeng, 2022), Under natural conditions, tree peony dormancy is broken after exposure to low winter temperatures. However, chilling treatment is ineffective in breaking *in vitro* shoot apical dormancy (Bouza et al., 1994). suggesting that the mechanisms of natural and *in vitro* dormancy may differ. At present, the molecular mechanisms underlying *in vitro* shoot apical dormancy in tree peony are poorly understood, with only two studies addressing this issue. Xu (2021) conducted a comprehensive analysis of the transcriptome, endogenous phytohormone concentrations, phenolic compound levels, carbohydrate contents, and enzyme activities in the leaves and stems of *in vitro* plantlets. In this study, key genes showing differential expression were screened, however, there was a lack of analysis of gene regulatory network structure. Similarly, Fu et al. (2021) carried out comparable work using peony stems and leaves. Nevertheless, both studies overlooked the potential influence of different sampling sites on the results and thus were unable to provide a precise elucidation of the mechanism underlying *in vitro* shoot apical dormancy in tree peony. Therefore, the regulatory network governing *in vitro* shoot dormancy in tree peony shoot apices needs to be further investigated.

*Paeonia* × *lemoinei* ‘High Noon’ is an inter-subsectional hybrid known for its ornamental value and stress resistance. It has strong potential for use as a potted flower, cut flower, scented tea, and landscaping plant. Although an efficient micropropagation protocol has been developed for this cultivar (Wen et al., 2016b, Wen et al., 2016c, Wen et al., 2018), *in vitro* shoot apical dormancy continues to limit its commercial propagation. To elucidate the molecular mechanisms underlying this dormancy, we used shoot apices from *Paeonia × lemoinei* ‘High Noon’ at four root induction stages (0, 10, 20, and 30 days) as experimental materials. First, we analyzed the endogenous hormone levels in the shoot apices during adventitious root formation. Then, we conducted transcriptome sequencing using Illumina HiSeq to identify dormancy-related genes and regulatory networks. This research provides a foundation for regulating shoot apical dormancy in *in vitro* tree peony plantlets and will contribute significantly to improving transplant survival rates—thus advancing the industrial development of the tree peony industry.

## 2 Materials and methods

### 2.1 Plant material

According to the rooting protocol we previously reported (Wen et al., 2016a, Wen et al., 2021), *in vitro* shoots of *Paeonia* × *lemoinei* ‘High Noon’ in good growth conditions were transferred to half-strength Murashige and Skoog medium (MS; Murashige and Skoog, 1962) with all microelements at half-strength. The medium contained 1.0 mg·L^-^¹ indole-3-butyric acid (IBA), 1.0 mg·L^-^¹ putrescine (PUT), 30.0 g·L^-^¹ sucrose, and 6.0 g·L^-^¹ agar. PUT was filter-sterilized using a 0.22 μm filter and added to the medium after autoclaving (120°C, 101 kPa, 20 min). The cultures were grown at 24 ± 1 °C in the dark for 30 days to induce rooting. During this process, the shoot apices entered a dormant state. Therefore, shoot apices (0.5 cm) were harvested at four stages (0d, 10d, 20d, 30d) of root induction and designated as R0, R10, R20, and R30. Each treatment was repeated three times. Samples were immediately frozen in liquid nitrogen and stored at -80°C for subsequent experiments.

### 2.2 Measurement of endogenous hormone content

The methods for extraction and purification of trans-Zeatin-riboside (ZR), gibberellin acid (GA_3_), indole-3-acetic acid (IAA), and abscisic acid (ABA) followed those described by Bollmark et al. (1988). Fresh tissue samples (0.3 g) were homogenized using a pre-chilled mortar with 10 mL of 80% methanol (v/v) extraction solution supplemented with 1 mM butylated hydroxytoluene as an antioxidant. Following a 4-hour incubation at 4°C, the homogenate was centrifuged at 4,000 rpm for 15 min at 4°C. The resulting supernatant was filtered through Chromosep C18 columns (C18 Sep-Park Cartridge, Waters Corp., Milford, MA), which had been preconditioned sequentially with 10 mL of absolute methanol and 5 mL of 80% methanol (v/v). The hormone fractions were eluted using 10 mL of absolute methanol followed by 10 mL of diethyl ether, then evaporated under nitrogen gas and reconstituted in 2 mL of phosphate-buffered saline (PBS, pH 7.5) containing 0.1% Tween 20 (v/v) and 0.1% gelatin (w/v) for ELISA analysis. The quantification of GA_3_, IAA, ZR, and ABA was performed using enzyme-linked immunosorbent assay (ELISA) kit, as described by (Yang et al., 2001). ELISA reagents—including mouse monoclonal antigens/antibodies against ZR, IAA, GA_3_, and ABA, and horseradish peroxidase (HRP)-conjugated IgG—were provided by the Phytohormones Research Institute (China Agricultural University). Color development in each well was detected using an ELISA Reader (model EL310, Bio-TEK, Winooski, VT) at an optical density of A490. The contents of ZR, IAA, GA_3_, and ABA were calculated according to Weiler et al. (1981), and all experiment were performed in triplicate.

### 2.3 RNA extraction, library construction, and sequencing

Total RNA was extracted from three biological replicates of shoot apices using the EASYspin Plus Plant RNA Kit (Aidlab, China). RNA integrity was assessed via 1.2% agarose gel electrophoresis, while RNA quantification and purity were determined using a Nanodrop spectrophotometer. After confirming RNA quality, cDNA library construction and transcriptome sequencing were conducted by Nuohe Zhiyuan Technology Co., Ltd. (Beijing, China). Library purification, PCR enrichment, and quality assessment were performed using the AMPure XP system. Sequencing was carried out on the Illumina HiSeq platform after successful library inspection. Additional quality control was performed using the Agilent Bioanalyzer 2100 system (Agilent Technologies, CA, USA) and the NanoPhotometer spectrophotometer (IMPLEN, CA, USA).

### 2.4 *De novo* assembly and unigene functional annotation

Clean reads were obtained by removing raw reads containing adapters, more than 10% poly-N, or low-quality sequences. The percentages of bases with Phred scores >20 (Q20) and >30 (Q30), GC content, and sequence duplication levels were calculated. Clean reads were assembled *de novo* into contigs using Trinity (Grabherr et al., 2011). Hierarchical clustering was performed using Corset (Nadia and Alicia, 2014) to obtain high-quality single-gene sequences were obtained. The assembled unigenes were annotated using BLASTx alignment (E-value < 1×10^−5^) to seven databases, including NCBI non-redundant protein (NR), nucleotide (NT), Pfam, Clusters of Orthologous Groups (KOG/COG), Swiss-Prot, Kyoto Encyclopedia of Genes and Genomes (KEGG), and Gene Ontology (GO) databases. Estscan (3.0.3) was used to decide the sequence direction of unigenes with no match in the database or marched unknown sequences.

### 2.5 Analysis of differentially expressed genes

The number of mapped clean reads for each unigene was quantified using RNA-seq by Expectation Maximization (RSEM) (Li and Dewey, 2011) and normalized into fragments per kilobase per million fragments (FPKM). Transcriptomes comparisons across experimental conditions were performed using the DESeq R package (v1.10.1). P-value adjustments were implemented following the Benjamini–Hochberg procedure to control the false discovery rate. Genes with P-value < 0.05 and absolute log2 fold-change > 1 were considered significantly differentially expressed. Gene Ontology (GO) enrichment analysis was conducted using the GOseq R package (Young et al., 2010), and KOBAS (Mao et al., 2005) was used to assess KEGG pathway enrichment. KEGG pathways with FDR ≤ 0.05 were considered significantly enriched. In addition to global DEG enrichment analysis, DEGs were also analyzed separately according to their upregulation or downregulation.

### 2.6 Gene expression validation by qRT-PCR

Total RNA derived from RNA sequencing was reverse-transcribed into cDNA using the Novozan HiScript II First Strand cDNA Synthesis Kit. Four genes with high expression levels and strong annotation support were randomly selected for validation. Primers were designed using Oligo 7 software (**Supplementary Table S1**) and synthesized by Shanghai Jierui Bioengineering Corporation. Primer specificity was verified using Novozan 2x Rapid Taq Master Mix. Real-time fluorescence quantitative PCR was performed using ChamQ Universal SYBR qPCR Master Mix as the fluorescent dye on a StepOne Real-Time PCR System (Applied Biosystems, Foster City, CA, USA). Each reaction was conducted using three biological replicates, each with three technical replicates. Relative gene expression levels were calculated using the 2^-ΔΔCT^ method and normalized to *Ubiquitin* as the internal reference gene (Wang et al., 2012).

### 2.7 Statistical analyses

The data were collated using Excel 2020 (Microsoft Corporation, Redmond, WA, USA). SPSS 26.0 (SPSS Inc., Chicago, IL, USA) was used for single-factor completely randomized statistical analysis of variance, and the LSD method was used to test the significant differences of the experimental data (*p* < 0.05). Figures were generated via Origin 2021 and Adobe Illustrator 2020 (Adobe Inc., San Jose, CA, USA).

## 3 Results

### 3.1 Hormone changes of shoot apices during adventitious root formation

**Figure 1:**
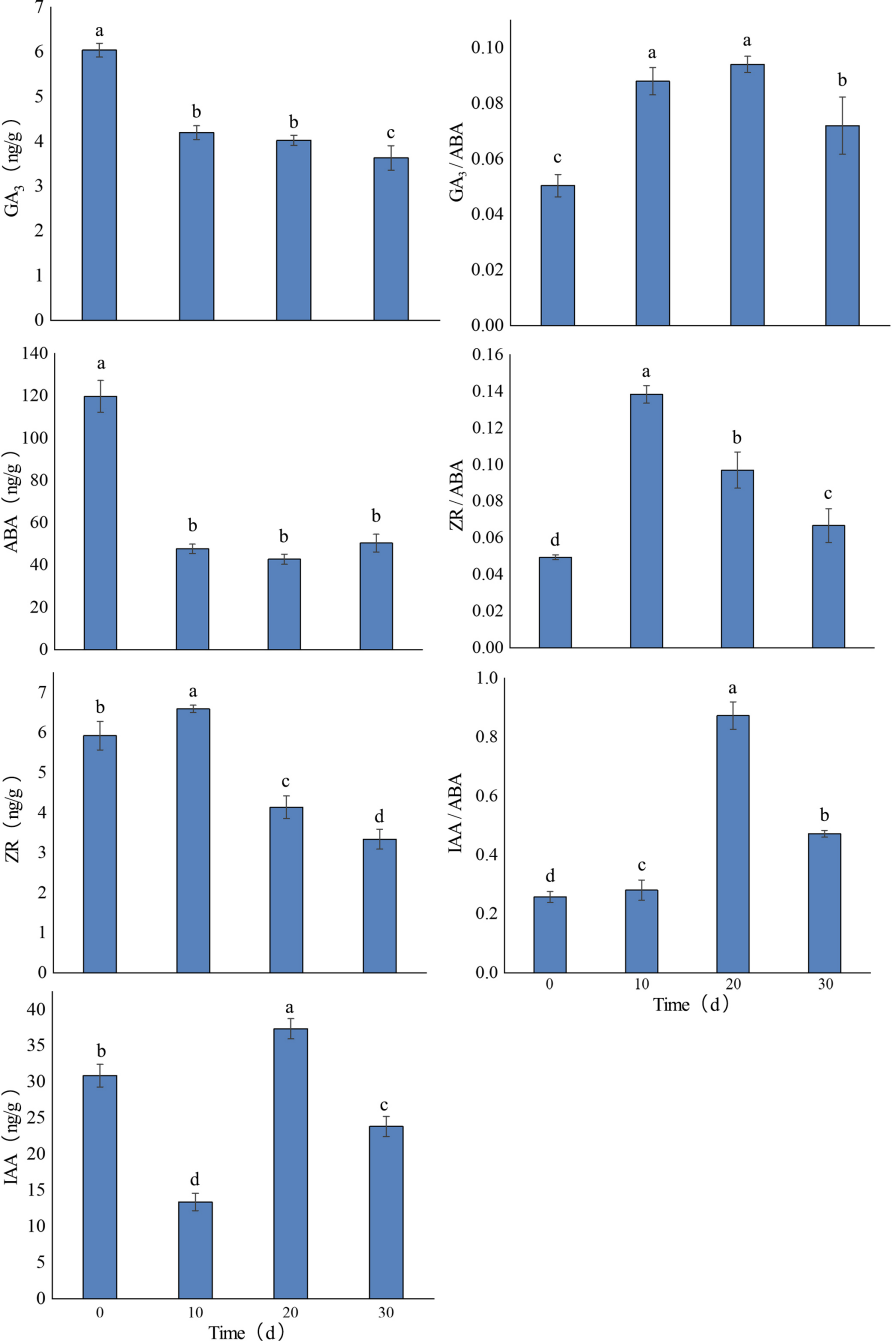
Changes in endogenous hormone levels in shoot apices during the *in vitro* rooting of *Paeonia* × *lemoinei* ‘High Noon’. Different letters indicate significant differences between samples at different rooting stages (p ≤ 0.05).

The contents of endogenous GA_3_, ABA, ZR, IAA, and their ratios to ABA varied significantly during *in vitro* rooting of *Paeonia* × *lemoinei* ‘High Noon’ (**Figure 1**). GA_3_ content decreased gradually and was negatively correlated with rooting progression. IAA content showed a trend of decreasing, then increasing, and finally decreasing, reaching a low of 13.40 ng/g on the 10th day and peaking at 37.37 ng/g on day 20. ZR content initially increased and then declined, peaking on the 10th day. In contrast, ABA content showed a sharp decline during the first 10 days but showed no significant difference between days 10 and 30 on rooting medium. The trends in the ratio of the three hormones (GA_3_, IAA, and ZR) to ABA first increased and then decreased; all ratios were higher than those at day zero during *in vitro* rooting (**Figure 1**). The ratios of GA_3_, IAA, and ZR to ABA first increased and then decreased, with all ratios higher than those at day 0. The GA_3_/ABA and IAA/ABA ratios peaked on day 20, while the ZR/IAA ratio peaked on day 10. These elevated hormone-to-ABA ratios during root culture stages may contribute to plantlet dormancy after rooting” for improved logical flow.

### 3.2 Analysis of transcriptome sequencing data and unigene functional annotation

**Figure 2:**
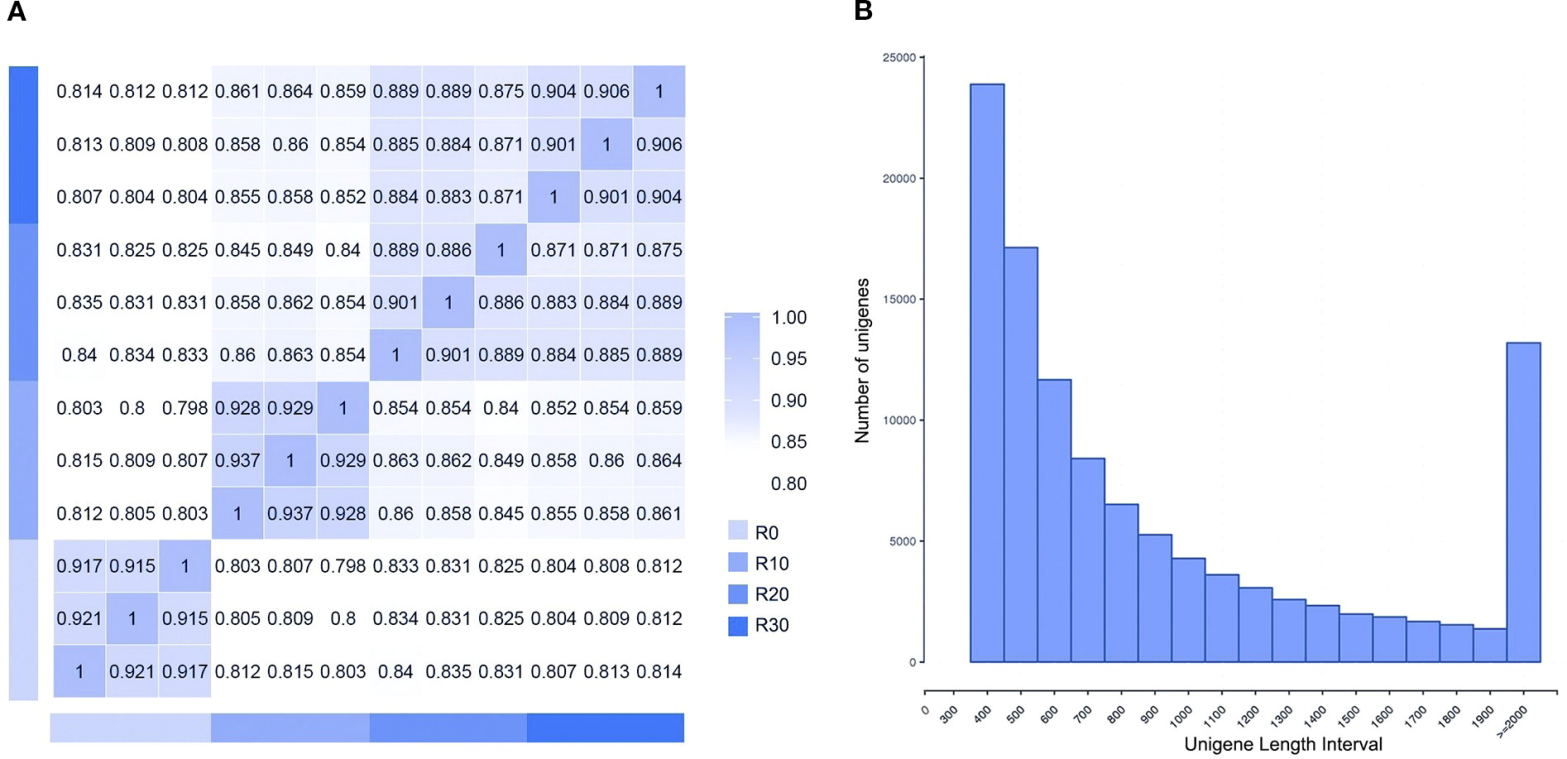
Sample sequencing results. **(A)** Sample correlation heat map. **(B)** Length distribution of assembled unigenes.

In this study, cDNA libraries were constructed from shoot apices of *Paeonia* × *lemoinei* ‘High Noon’ at 0, 10, 20, and 30 days during adventitious root formation. Approximately 7G of clean reads were obtained, with an error rate of 0.03%. Q20 and Q30 scores reached at least 96.6% and 91.3%, respectively. A total of 263,543 contigs ≥200 bp were obtained and further assembled into 121,843 single genes. The average unigene length was 1,003 bp, with an N50 of 1,387 bp. Data from the same sampling time points exhibited good reproducibility (**Figure 2A**). As shown in **Figure 2B**, there were 77,000 unigenes with length ≥ 500 bp and 12,525 unigenes with length ≥ 2000 bp, with the highest proportion (36.8%) between 301 and 500 bp—indicating that the data were of high quality for downstream analysis.

Using BLASTx analysis, 85,174 unigenes (69.9%) were annotated against seven databases (NR, NT, KEGG, SwissProt, PFAM, GO, and COG/KOG) (**Table 1**). Based on the annotation information from the NR database, GO functional annotation was conducted using Blast2GO (**Supplementary Figure S1A**; **Supplementary Table S3**), assigning 60,033 unigenes to 56 branches across three main categories: biological processes, molecular functions, and cellular components with. Comparison with the KEGG database annotated 19,390 unigenes to 132 pathways across five categories and 19 branches (**Supplementary Figure S1**; **Supplementary Table S4**).

**Table 1:** Statistics of unigene function annotation.

Annotation	Number of Genes	Percentage (%)
NR	53,311	43.75
NT	63,453	52.07
KEGG	19,390	15.91
SwissProt	56,022	45.97
PFAM	60,033	49.27
GO	60,033	49.27
COG/KOG	17,139	14.06
All Databases	8,810	7.23
At least one Database	85,174	69.9
In NR, NT, PFAM, GO and COG/KOG	12,255	10.06
Total Unigenes	121,843	100

### 3.3 Analysis of DEGs at different stages of rooting induction

**Figure 3:**
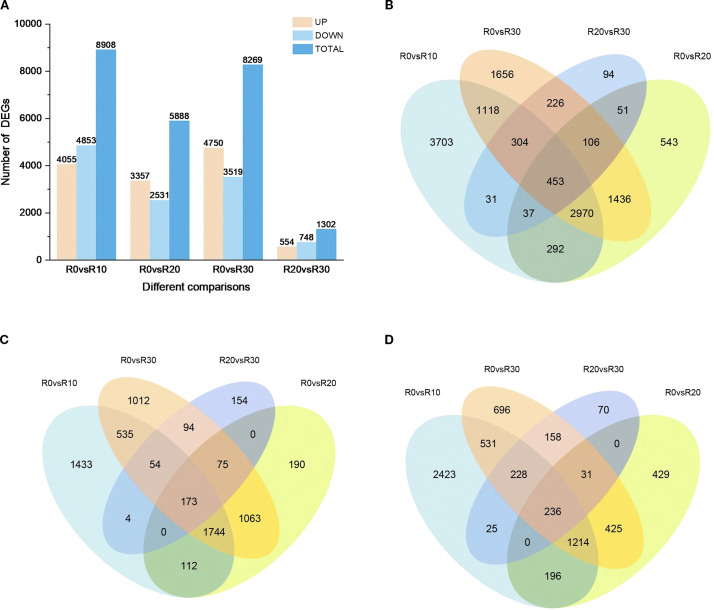
DEGs at different stages of rooting induction. **(A)** Number of upregulated and downregulated DEGs at each stage of rooting induction. **(B)** Venn diagram of DEGs across dormancy stages. **(C)** Venn diagram of upregulated DEGs during different dormancy stages. **(D)** Venn diagram of downregulated DEGs during different dormancy stages.

To explore differentially expressed genes (DEGs) involved in shoot apical dormancy, 13,020 DEGs were identified across four comparison groups: R0 vs R10, R0 vs R20, R0 vs R30, and R20 vs R30 (adjusted P < 0.05 and |log2FoldChange| >1 (**Figure 3**). The largest number of DEGs (8,908) occurred in R0 vs R10 comparison and the number of DEGs in R20 vs R30 comparison (1,302) was lowest, indicating that the *in vitro* shoot apical entered dormancy at 30 days of root induction, and gene expression began to stabilize. Notably, 54.48% of DEGs were downregulated in R0 vs R10, while 42.99% and 42.56% were downregulated in R0 vs R20 and R0 vs R30, respectively, indicating suppression of shoot apical activity around day 10 (**Figure 3A**). Across all comparisons, 453 DEGs were expressed in all four stages (**Figure 3B**), including 173 upregulated (**Figure 3C**) and 236 downregulated genes (**Figure 3D**). In comparison between R0 and R10, 3,703 distinctive DEGs were observed, while fraction of unique DEGs were identified in R0 vs R20 (543), R0 vs R30 (1656) and R20vsR30 (94) comparisons, indicating that genes undergo drastic transcriptional level changes in the stem tip tissue at 10 days of root induction.

### 3.4 Functional classification and enrichment of DEGs

**Figure 4:**
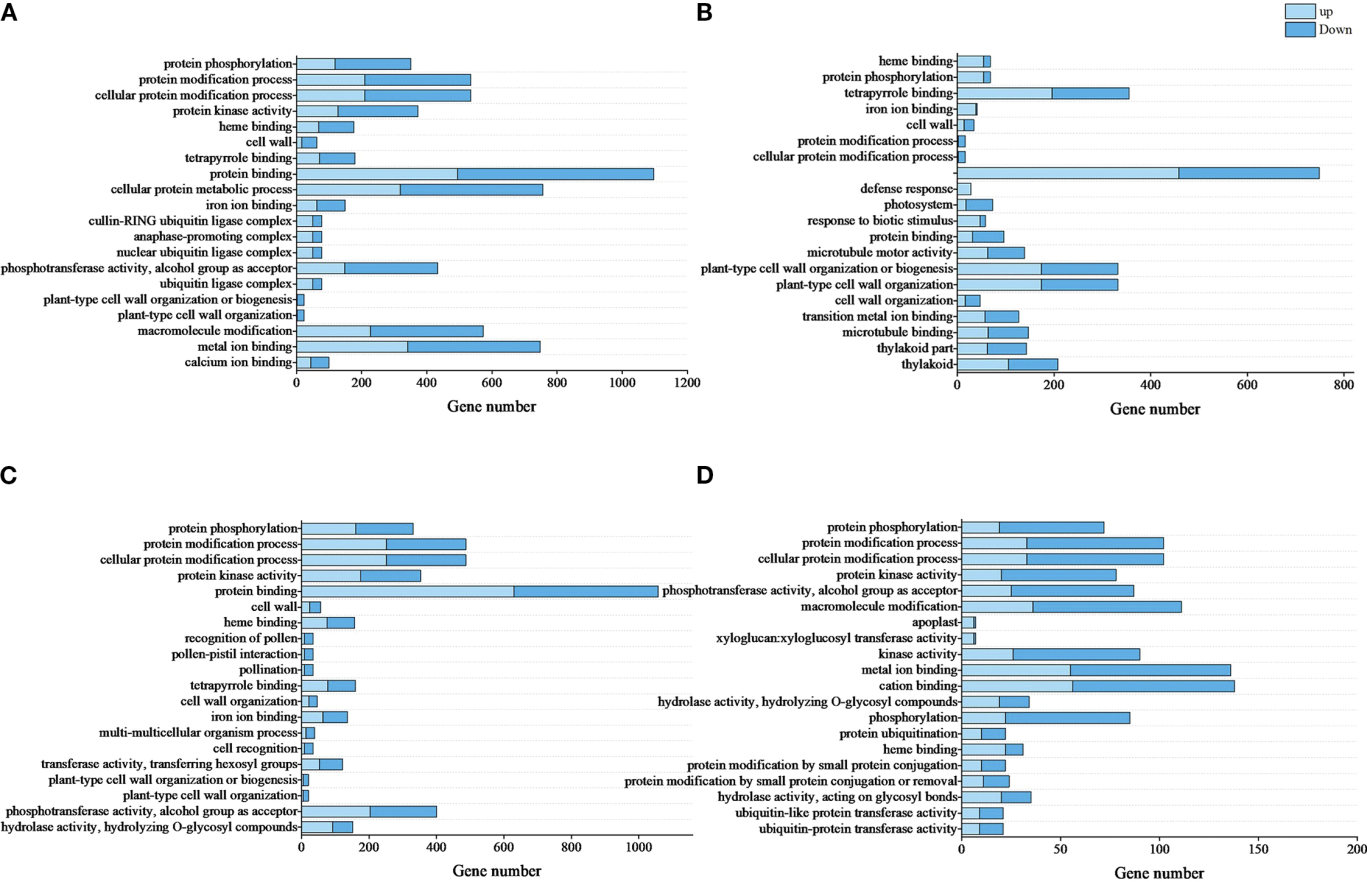
Top 20 enriched GO terms of DEGs. **(A)**, R0 vs R10, **(B)**, R0 vs R20, **(C)**, R0 vs R30, **(D)**, R20 vs R30. Light blue bars represent upregulated DEGs, and dark blue bars represent downregulated DEGs. GO terms are listed in ascending order of -log_10_Qvalue results. The top term had the highest significance.

Through GO enrichment analysis revealed significant alterations in biological functions related to *in vitro* shoot apical dormancy during root formation (**Figure 4**). In R0 vs R10, DEGs were enriched in protein modification, metabolism, and cell wall formation (biological processes); cell wall and ubiquitin ligase complex (cellular components); and protein kinase activity, heme binding, and protein binding (molecular functions)—mostly dominated by downregulated genes (**Figure 4A**; **Supplementary Table S5**). In R0 vs R20, DEGs were enriched in the molecular function subclass of heme binding, and the biological process subclass of protein phosphorylation to the highest extent, and DEGs were enriched in the defense response and biotic stimulus response, which were dominated by upregulated genes (**Figure 4B**; **Supplementary Table S6**). There was a certain similarity in the enriched terms between R0 vs R30 and R0 vs R10. Both comparisons were enriched highly in the functional branches of protein modification such as protein phosphorylation but less in cellular components, dominated by upregulated genes (**Figure 4C**; **Supplementary Table S7**). Further comparison between R20 and R30 revealed that DEGs were significantly reduced compared to the other three groups, and most of them were downregulated (**Figure 4D**; **Supplementary Table S8**).

**Figure 5:**
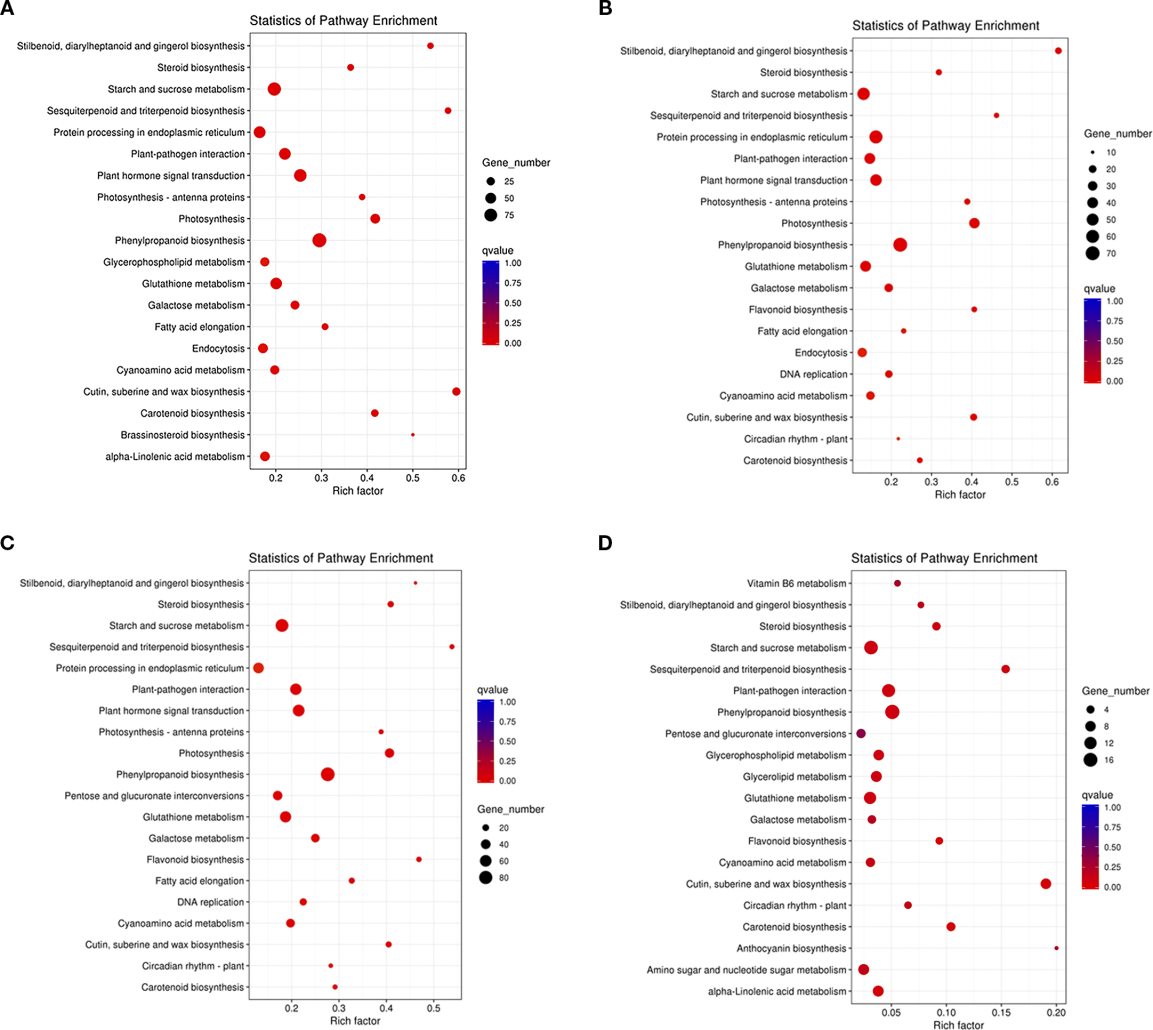
Top 20 KEGG pathways enriched for DEGs related to *in vitro* shoot apical dormancy. **(A)** R0 vs R10, **(B)** R0 vs R20, **(C)** R0 vs R30, **(D)** R20 vs R30. The Y-axis represents KEGG pathways; the X-axis shows the enrichment factor. The color of the dot corresponded to a different q-value. q-value (red = more significant), and dot size indicates the number of DEGs in each pathway.

By KEGG enrichment analysis showed that DEGs in R0 vs R10, R0 vs R20, R0 vs R30, and R20 vs R30 were enriched in 111, 101, 112, and 79 pathways, respectively. We found that the major enriched pathways were essentially the same (**Figure 5**). The DEGs of R0 vs R10, R0 vs R20, and R0 vs R30 had the highest enrichment in the phenylpropanoid biosynthesis, followed by higher enrichment in the photosynthesis, plant hormone signal transduction, cutin, suberine and wax biosynthesis, starch and sucrose metabolism, and plant–pathogen interaction. pathways. Comparing the R20 and R30 groups showed that DEGs were mainly enriched in cuticles; suberine and wax biosynthesis; phenylpropanoid biosynthesis; plant-pathogen interaction; carotenoid biosynthesis; terpene biosynthesis; and starch and sucrose metabolism pathways. There were significant differences in the number of DEGs enriched in the same pathway at different periods of root induction. At 10, 20, and 30 days of root induction, 93, 70, and 87 DEGs were enriched in the phenylpropanoid biosynthesis pathway, while 72, 46, and 61 DEGs were enriched in plant hormone signal transduction. These findings suggest that plant hormone signaling, phenylpropanoid biosynthesis, starch and sucrose metabolism, and pathogen response pathways play key roles *in* shoot apical dormancy during adventitious root formation.

### 3.5 Screening of key dormancy-associated DEGs

**Figure 6:**
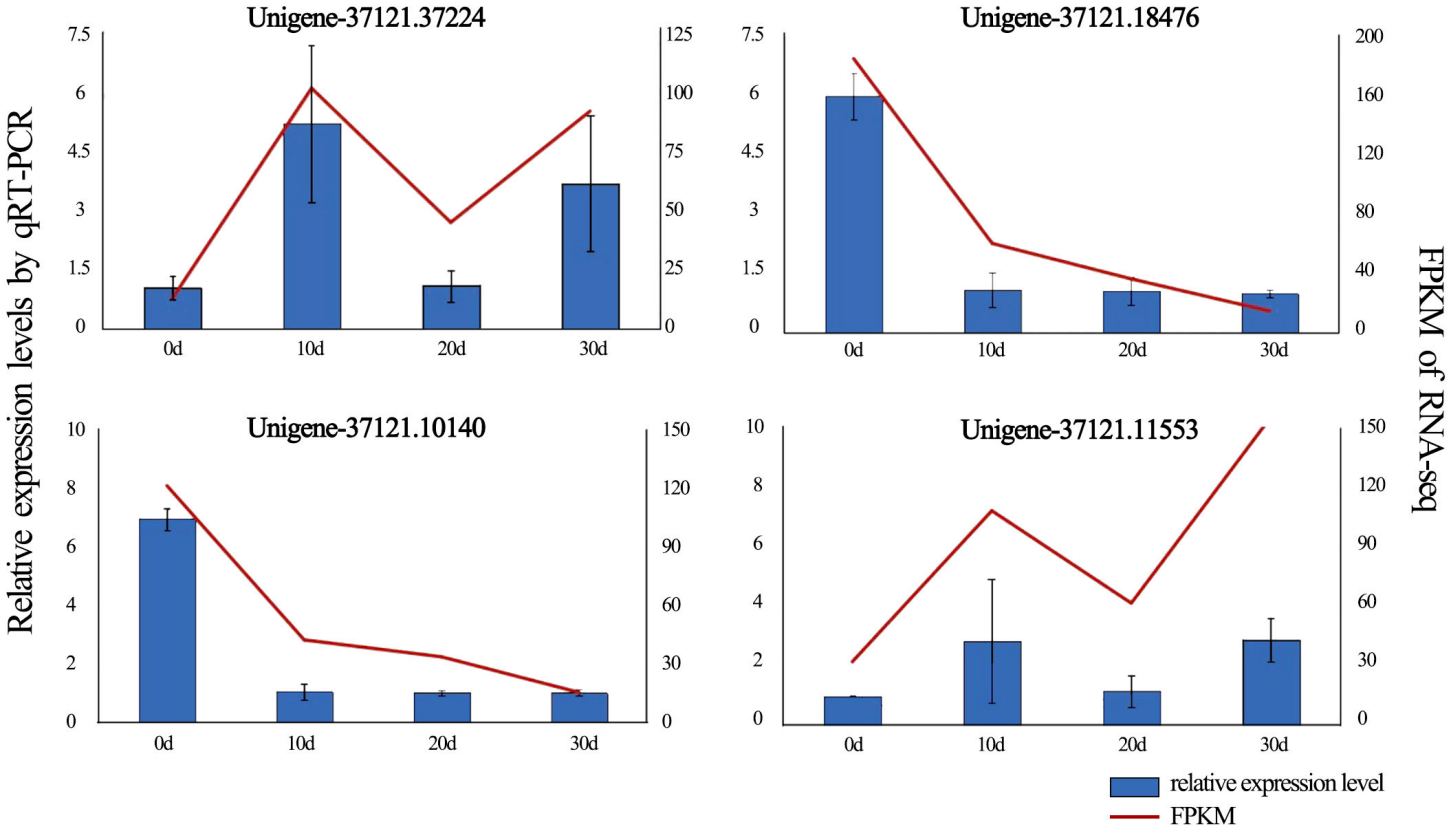
qRT-PCR confirmed DEG expression levels, with error bars showing standard deviation from triplicate replicates. Expression patterns of four DEGs associated with *in vitro* shoot apical dormancy were detected by qRT-PCR (blue bars) and RNA-Seq (red lines).

**Figure 7:**
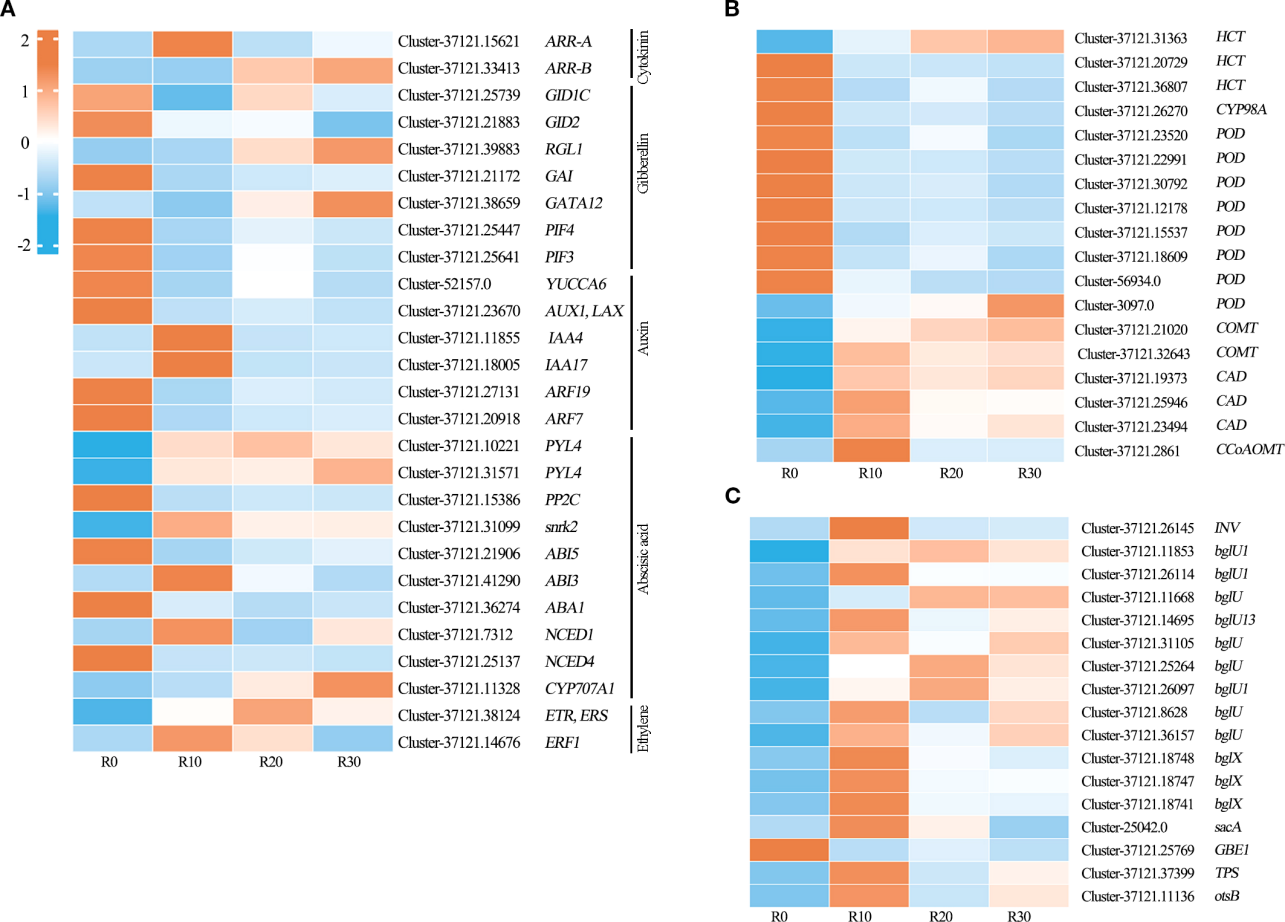
Heatmap of Z-score–normalized RPKM values for DEGs in significantly enriched pathways during shoot apical dormancy, including **(A)** plant hormone signal transduction, **(B)** phenylpropanoid biosynthesis, and **(C)** starch and sucrose metabolism **(C)** during shoot apical dormancy period. Heatmap colors represent RPKM levels: red for high expression, blue for low expression.

Based on KEGG analysis and expression profiling of related pathways, a total of 62 shoot apical dormancy-associated DEGs were identified in this study (**Figure 6**), including 27 DEGs related to plant hormone signaling, 18 in phenylpropanoid biosynthesis, and 17 in starch and sucrose metabolism. Among them, DEGs in the plant hormone signal transduction pathway were mainly involved in cytokinin (CTKs), gibberellin (GA), auxin (IAA), abscisic acid (ABA), and ethylene (ET) signaling pathways (**Figure 7A**). *ARR-A* and *ARR-B* were identified from the cytokinin signal transduction pathway and encoded two-component response regulators of the ARR family. The gibberellin signaling pathway included the receptor gene *GID1*, DELLA protein gene RGL1, GAI, GATA transcription factor *GATA12*, F-box protein gene *GID2*, and transcription factors *PIF3* and *PIF4*. Six DEGs in the auxin signal transduction pathway were identified, including *YUCCA6* encoding indole-3-pyruvate monooxygenase (YUC), *AUX1* encoding auxin influx carrier protein (AUX1), *IAA4* and *IAA17* encoding auxin-responsive proteins (AUX/IAA), and *ARF7* and *ARF19* encoding ARF transcription factors. Ten DEGs were identified from ABA synthesis, signal transduction, and metabolic pathways, including zeaxanthin epoxidase gene *ABA1*; 9-cis-epoxycarotenoid dioxygenase genes *NCED1* and *NCED4* in ABA biosynthesis; 8’-hydroxylase gene *CYP707A1* in ABA catabolism; as well as ABA receptor gene *PYL4*, protein phosphatase gene *PP2C*, serine/threonine protein kinase gene *SnRK2*, and ABA response element-binding factor genes *ABI5* and *ABI3* in the ABA signaling pathway. Two key genes were identified in the ethylene signal transduction pathway: *ETR* encoding the ethylene receptor and *ERF1* encoding the ethylene-responsive transcription factor. *POD*, *COMT*, *CAD*, *CCoAOMT*, *PAL*, *4CL*, and *CCR* genes were involved in the phenylpropanoid biosynthetic pathway (**Figure 7B**). The DEGs identified in the starch and sucrose metabolism pathway included β-fructofuranosidase gene *INV*, beta-glucosidase gene *BGLU/BGLX*, sucrose synthase gene *sacA*, 1,4-α-glucan branching enzyme gene *GBE1*, and phosphatase genes *TPS* and *otsB* (**Figure 7C**).

### 3.6 Validation of RNA-seq by qRT-PCR

To validate the reliability of the RNA-seq results, four DEGs (Unigene-37121.37224, Unigene-37121.18476, Unigene-37121.10140, Unigene-37121.11553) with high expression and rich annotation information were randomly selected for qRT-PCR validation during *in vitro* shoot apical dormancy (**Figure 6**). The expression levels of Unigene-37121.18476 and Unigene-37121.10140 were significantly downregulated after rooting compared with before rooting, while the remaining two DEGs were significantly upregulated. Although the fold changes obtained from qRT-PCR did not exactly match the FPKM values from transcriptome sequencing, they showed the same expression trends, indicating that the RNA-seq data were accurate and reliable.

## 4 Discussion

Plant dormancy is regulated by multiple factors, including endogenous hormones, carbohydrates, and phenolic acids, acting in a coordinated manner (Rohde and Bhalerao, 2007). Among these, changes in endogenous hormone levels are key factors influencing tree peony dormancy (Zheng et al., 2009; Zhang et al., 2018b, Zhang et al., 2020). Specifically, trans-Zeatin-riboside (ZR), gibberellin acid (GA_3_), indole-3-acetic acid (IAA), and abscisic acid (ABA) interact synergistically to regulate the dormancy in tree peony (Fu et al., 2021; Xu, 2021).

### 4.1 Regulation mechanism of endogenous ZR

Studies have shown that ZR facilitator the release of tree peony dormancy (Zheng et al., 2009; Liu et al., 2004). In this study, the ZR content increased rapidly during days 0 to10, then decreased significantly and remained low during days 10 to 30 of root induction. These results were consistent with previous studies in tree peony (Fu et al., 2021). Therefore, a reduction in endogenous ZR is critical for inducing dormancy in the apical shoot of *in vitro* tree peony plantlets.

**Figure 8:**
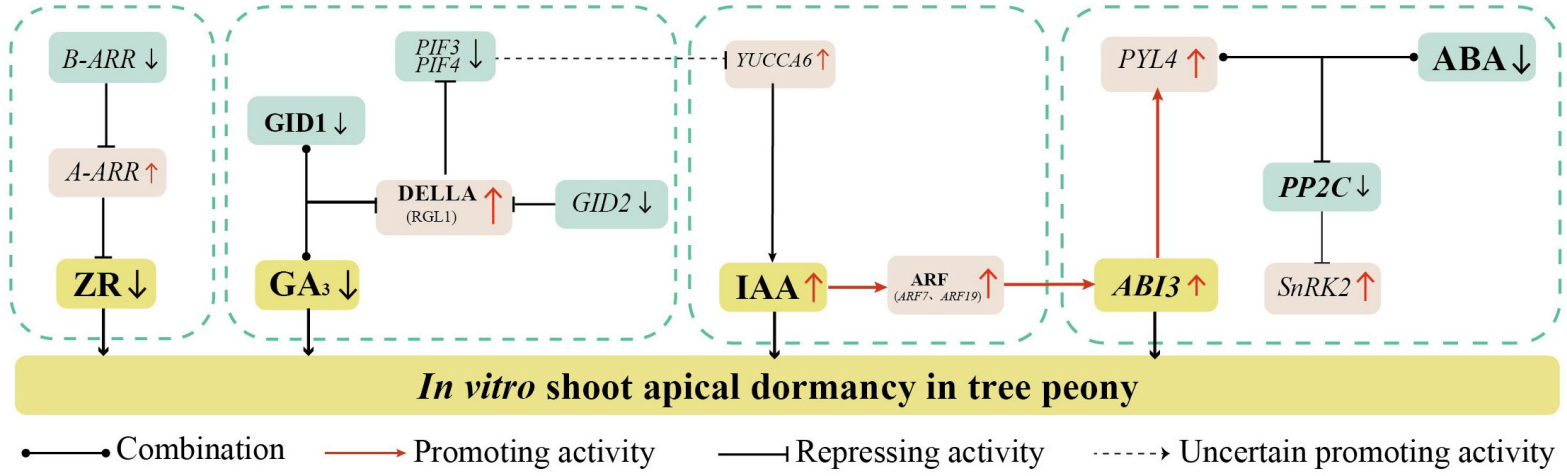
Hypothetical model of the endogenous hormone regulatory networks underlying *in vitro* shoot apical dormancy in *Paeonia* × *lemoinei* ‘High Noon’.

Recent studies have reported molecular mechanisms by which ZR regulates dormancy release. In *Arabidopsis*, type-B Arabidopsis response regulator (*B-ARR*) activate the transcription of type-A response regulator (*A-ARR*) which acts as a negative regulator of CTK (Hutchison et al., 2006; Punwani et al., 2010; Punwani and Kieber, 2010). This regulatory mode has also been observed in *Zea mays* (Zeng et al., 2021), *Oryza sativa* (Gao et al., 2014), and *Glycine max* (Le et al., 2011). In this study, *A-ARR* expression was significantly upregulated, while *B-ARR* expression was significantly downregulated during days 10 to 30 of root induction. Therefore, we speculate that the upregulation of A*-ARR* may be associated with suppression of *B-ARR* transcription (**Figure 8**). This regulatory cascade may reduce ZR content in shoot apices, ultimately inducing shoot apical dormancy.

### 4.2 Regulation mechanism of endogenous GA_3_

Previous studies have shown that high level of GA_3_ levels promote dormancy release during chilling in tree peony, while low GA_3_ levels induce dormancy (Zhang et al., 2021; Zheng et al., 2009; Niu et al., 2025). In this study, the GA_3_ content decreased rapidly after transfer to the rooting medium and remained low, which was consistent with Fu et al. (2021). This suggests that the maintained low levels of GA_3_ are associated with the dormancy state.

Currently, a large number of studies have explored the molecular mechanisms of GA in bud dormancy under natural conditions. The classical GA-GID1-DELLA module mediates GA signaling, with GA perception dependent on GA-INSENSITIVE DWARF1 (GID1). Upon binding GA, GID1 interacts with DELLA proteins to form the GA-GID1-DELLA complex (Locascio et al., 2013; Liu et al., 2015). Transcriptome-based analysis in this study suggests that reduced GA_3_ in the apical shoot may inhibit GA-GID1 binding, upregulating DELLA protein gene RGL1, consistent with the dormancy model by Gao et al. (2023). Meanwhile sustained *GID2* reduction may weaken DELLA protein ubiquitination, thereby inhibiting *PIF3* and *PIF4* transcription (Zhang et al., 2023) (**Figure 8**). This suppresses downstream GA pathway genes, maintaining shoot apex dormancy.

### 4.3 Regulation mechanism of endogenous IAA

Studies have shown that IAA and ABA act synergistically to inhibit seed germination, and exogenous application of IAA effectively enhances plant dormancy (Liu et al., 2013; Shuai et al., 2017). It was shown that exogenous IBA significantly increased the content of endogenous IAA (Wang et al., 2014). In this study, the rooting medium was supplemented with IBA, which promoted IAA accumulation in the *in vitro* plantlets. Ultimately, the accumulation of IAA in the apical shoots promoted *in vitro* shoot apical dormancy in tree peony.

It has been shown that the YUCCA gene family maintains seed dormancy by encoding flavin monooxygenase to regulate the synthesis of IAA (Liu et al., 2017). When auxin levels are high, auxin-responsive transcription factors *ARF10* and *ARF16* are released to activate *ABI3* transcription, thereby enhancing ABA signaling transduction without increasing ABA levels. This contributes to the maintenance of seed dormancy (Liu et al., 2013). In this study, after 20 days of root induction, the IAA content was significantly upregulated and reached its peak. The expression of auxin response factor *ARF7* and *ARF9* was significantly upregulated as the IAA content increased. It might have promoted *ABI3* transcription to activate the ABA signaling pathway, and ultimately induced *in vitro* shoot apical dormancy in tree peony. The result was also consistent with Fu et al. (2021) and Pan et al. (2025). Notably, Sun et al. (2013) found that *PIF4* increased IAA content by activating the expression of auxin synthesis genes in a dark environment. Meanwhile, the expression of *PIF3* and *PIF4* were downregulated in the early stage of root induction, which negatively regulated the key gene *YUCCA6* for IAA synthesis, then in turn affected the accumulation of IAA to promote shoot apical dormancy.

### 4.4 Regulation mechanism of endogenous ABA

A high level of ABA content plays a dominant role in establishing bud dormancy in tree peony under natural conditions, and dormancy release mainly depends on the GA/ABA ratio (Mornya and Cheng, 2013). In *in vitro* tree peony plantlets, Fu et al. (2021) also suggested that high levels of ABA content determine dormancy. However, in contrast to previous research, our study observed a sharp decrease in ABA content during the root induction process of *in vitro* tree peony plantlets. We attribute this phenomenon to two main factors. First, our experimental material consisted of shoot apices, whereas previous studies used stem and leaf tissues. Second, we employed a two-step rooting method in which samples were frozen during the first 0 – 8 days of root induction. The initially high ABA level may be related to enhanced resistance to low-temperature stress (Wang et al., 2019). Therefore, we propose that ABA levels are not the dominant factor inducing dormancy in *in vitro* tree peony plantlets.

Recent studies have revealed complex regulatory interactions among ABA, IAA, and GA signaling pathways. The PYR/PYL/RCAR–PP2C–SnRK2 cascade is the core ABA-mediated signaling network, where ABA directly acts on the negative regulator PP2C phosphatase and the positive regulator SnRK2 (Ma et al., 2009; Park et al., 2009). PYL could positively regulate the plant responses to extreme temperature through inducing the expression of downstream genes in *Arabidopsis* (Zhang et al., 2019). In this study, the expression of PYL4 was significantly upregulated during the 0–10 day period of root induction. It is likely that ABA bound to PYR/PYL receptors during this period, leading to increased expression of resistance-related genes and enhanced plant resistance to low-temperature stress. Sun et al. (2013) found that *PIF4* increases IAA levels by activating the expression of auxin synthesis genes in dark environments. In our study, however, the expression levels of *PIF3* and *PIF4* were downregulated at the early stage of root induction. This downregulation may have negatively regulated the key auxin biosynthesis gene *YUCCA6*, thereby modulating IAA accumulation. As IAA levels increased, auxin-responsive transcription factors *ARF10* and *ARF16* were released, which in turn activated *ABI3* transcription. This activation likely promoted *in vitro* shoot apical dormancy in tree peony. Therefore, we speculate that during the 10–30 day period of root induction, GA_3,_ and IAA synergistically activate the ABA signaling pathway, which plays a regulatory role in inducing shoot apical dormancy in *in vitro* tree peony plantlets (**Figure 8**).

## 5 Conclusion

In this study, *Paeonia × lemoinei* ‘High Noon’ shoot apices were used as the experimental material to investigate the mechanisms by which endogenous hormones affect *in vitro* shoot apical dormancy in tree peony. ZR and GA_3_ were identified as key hormones regulating apical shoot dormancy, and the accumulation of IAA in shoot apices promoted dormancy. ABA was not found to be a determinant of shoot apical dormancy, but GA_3_ and IAA may synergize with ABA to regulate dormancy in tree peony.

Transcriptome analyses revealed 27 key dormancy-regulated genes in the plant hormone pathway, including *ABA1*, *NCED1*, *CYP707A1*, *PYL4*, *PP2C*, RGL1, *GATA12*, *YUCCA6*, *ARF7*, *ARF19*, *ERF1*, and others. Eighteen genes, including *POD*, *COMT*, *CAD*, *HCT*, etc., were identified in the phenylpropane biosynthetic pathway. Seventeen genes, including *INV*, *BGLU*, *BGLX*, *sacA*, *GBE1, TPS*, and others, were identified in the starch and sucrose metabolic pathways. Based on these findings, a regulatory network for shoot apical dormancy in tree peony centered on endogenous ZR and GA_3_ was established.

In summary, this study comprehensively explored the mechanism of endogenous hormone regulation of shoot apical dormancy at multiple levels. The results contribute to a comprehensive understanding of the molecular mechanisms underlying shoot apical dormancy and provide a valuable theoretical basis and practical guidance for improving the transplanting survival rate of micropropagation, thereby promoting industrial application of this technology in tree peony.

This study only covered the exploration of relevant genes involved in the regulation of shoot apical dormancy by endogenous hormones. In the future, key genes identified in this study can be further investigated using model plants or through the establishment of a genetic transformation system in tree peony, thereby providing deeper insight into the molecular mechanisms underlying *in vitro* shoot apical dormancy.

## Data Availability

The data presented in the study are deposited in the NCBI repository, accession number PRJNA1274627.
