# Supplementary material for: Analysis of endogenous hormones and transcriptomes involved in in vitro shoot apical dormancy during adventitious root formation in tree peony
Source: Front Plant Sci. 2025 Sep 11;16:1610747. doi: 10.3389/fpls.2025.1610747 (PMC12462052; doi:10.3389/fpls.2025.1610747)
Supplement: Supplementary file 1 [file DataSheet2.docx]

Supplementary Material

# Supplementary Figures

#
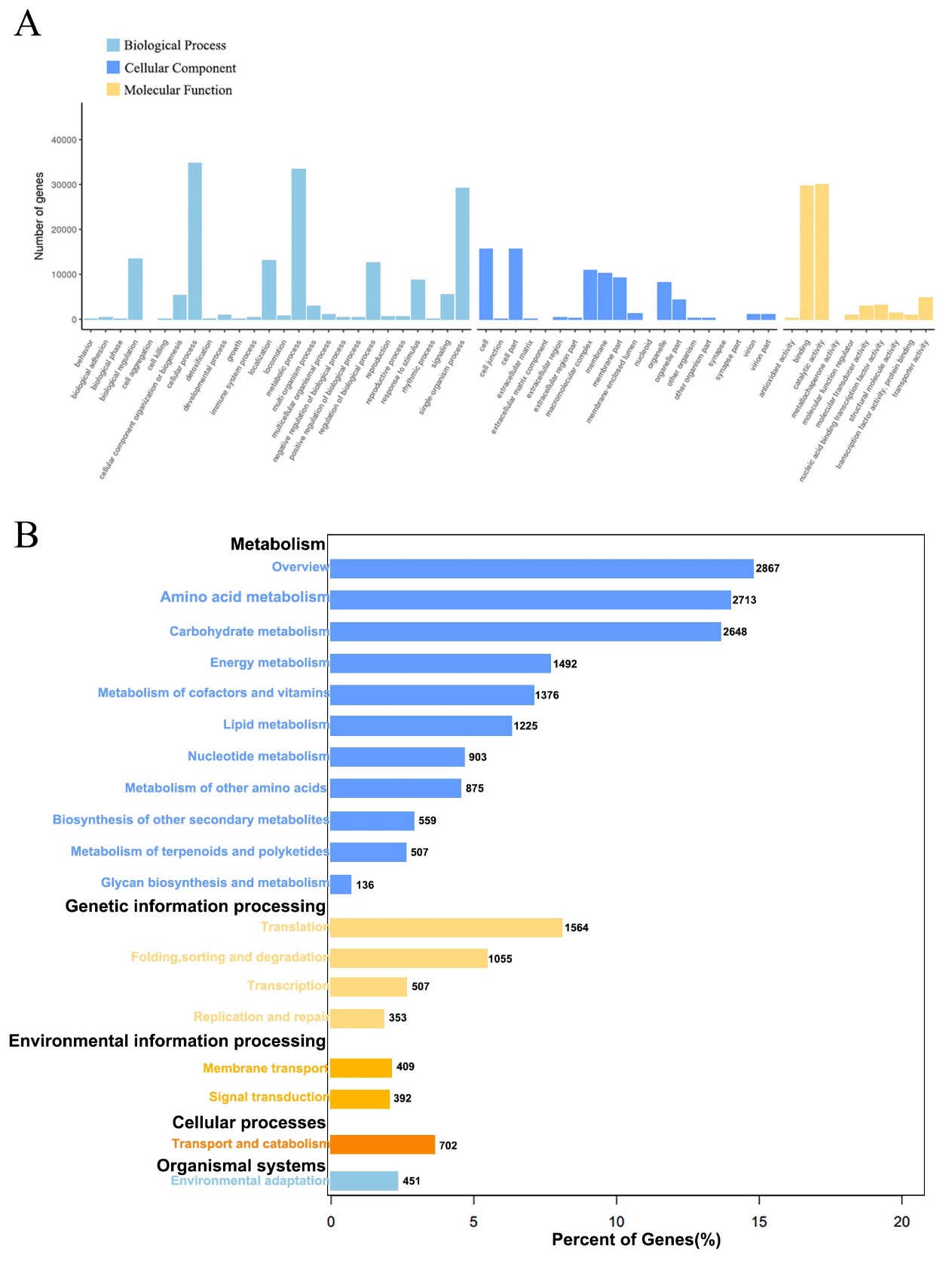


**Supplementary Figure 1.** Functional annotation and classification of GO and KEGG. (A) GO classification of unigenes. The unigenes corresponded to three main categories: biological process, cellular component, and molecular function. Y-axis indicated the number of annotated unigenes in a category and X-axis represented GO terms. (B) KEGG pathway annotation of unigenes. Y-axis represented enriched pathways and X-axis represented the number and the percent of annotated unigenes.

# Supplementary Tables

# Supplementary Table S1. Primer sequences for the quantification of transcripts by qRT-PCR.

| Gene ID | Gene name | Forward primer sequence (5'→3') | Reverse primer sequence (5'→3') |
| --- | --- | --- | --- |
| Unigene-37121.37224 | *EXOC7* | CTCCACATCTTCAGGGGCAAAC | GGAACGTCGTGACGGCGTAT |
| Unigene-37121.18476 | *GERD* | TGATTGTTCACATTGCGATAGG | CTTGAGCCATTAGCCGTCCC |
| Unigene-37121.10140 | -- | CTTGAAATAGAGGTAGCAATCCAC | AGGCACCGACACTAGCATAA |
| Unigene-37121.11553 | -- | GTTCTTATCGGATTTATCGGTTCG | GTAGCAAAGTTCATGGATTGGTGA |
| -- | *Ubiquitin* | GACCTATACCAAGCCGAAG | CGTTCCAGCACCACAATC |

# Supplementary Table S2. Statistics assembly for transcript and unigene in transcriptome.

|  | Transcript number | Unigene number |
| --- | --- | --- |
| N50 | 1615 | 1387 |
| N90 | 470 | 440 |
| Min Length | 301 | 301 |
| Mean Length | 1113 | 1003 |
| Median Length | 721 | 619 |
| Max Length | 117803 | 117803 |

# Supplementary Table S3. Statistics of GO function annotation results of unigenes.

| Function | Functional category | Unigene number |
| --- | --- | --- |
| Biological Process | biological phase | 47 |
|  | cellular component organization or biogenesis | 5428 |
|  | regulation of biological process | 12681 |
|  | biological adhesion | 449 |
|  | rhythmic process | 37 |
|  | multi-organism process | 3000 |
|  | reproductive process | 548 |
|  | immune system process | 346 |
|  | single-organism process | 29086 |
|  | detoxification | 51 |
|  | positive regulation of biological process | 494 |
|  | localization | 13153 |
|  | multicellular organismal process | 1051 |
|  | behavior | 89 |
|  | locomotion | 746 |
|  | cellular process | 34792 |
|  | developmental process | 905 |
|  | metabolic process | 33392 |
|  | signaling | 5484 |
|  | cell killing | 53 |
|  | growth | 54 |
|  | cell aggregation | 2 |
|  | reproduction | 619 |
|  | biological regulation | 13461 |
|  | response to stimulus | 8795 |
| Molecular Function | transcription factor activity, protein binding | 1037 |
|  | molecular transducer activity | 2920 |
|  | transporter activity | 4794 |
|  | antioxidant activity | 355 |
|  | molecular function regulator | 900 |
|  | binding | 29714 |
|  | structural molecule activity | 1479 |
|  | nucleic acid binding transcription factor activity | 3234 |
|  | catalytic activity | 30002 |
|  | metallochaperone activity | 10 |
|  | negative regulation of biological process | 504 |
| Cellular Component | membrane | 10243 |
|  | other organism | 294 |
|  | membrane-enclosed lumen | 1264 |
|  | extracellular matrix component | 3 |
|  | virion part | 1185 |
|  | extracellular region | 395 |
|  | cell part | 15675 |
|  | cell | 15675 |
|  | synapse part | 5 |
|  | extracellular matrix | 137 |
|  | synapse | 5 |
|  | organelle | 8173 |
|  | other organism part | 294 |
|  | macromolecular complex | 10952 |
|  | extracellular region part | 367 |
|  | organelle part | 4382 |
|  | nucleoid | 3 |
|  | cell junction | 40 |
|  | membrane part | 9259 |
|  | virion | 1185 |

# Supplementary Table S4. Unigene enrichment accounted for more than 1% of pathways.

| KEGG Pathway | Pathway ID | Gene Number (proportion) |
| --- | --- | --- |
| Carbon metabolism | ko01200 | 1593(8.22%) |
| Biosynthesis of amino acids | ko01230 | 1530(7.89%) |
| Purine metabolism | ko00230 | 648(3.34%) |
| Ribosome | ko03010 | 639(3.30%) |
| Valine, leucine and isoleucine degradation | ko00280 | 587(3.03%) |
| Glyoxylate and dicarboxylate metabolism | ko00630 | 553(2.85%) |
| Glycolysis / Gluconeogenesis | ko00010 | 541(2.79%) |
| Oxidative phosphorylation | ko00190 | 540(2.78%) |
| Pyruvate metabolism | ko00620 | 537(2.77%) |
| Fatty acid metabolism | ko01212 | 496(2.56%) |
| Pyrimidine metabolism | ko00240 | 459(2.37%) |
| 2-Oxocarboxylic acid metabolism | ko01210 | 457(2.36%) |
| Glycine, serine and threonine metabolism | ko00260 | 455(2.35%) |
| Alanine, aspartate and glutamate metabolism | ko00250 | 416(2.15%) |
| Aminoacyl-tRNA biosynthesis | ko00970 | 412(2.12%) |
| Starch and sucrose metabolism | ko00500 | 412(2.12%) |
| ABC transporters | ko02010 | 409(2.11%) |
| Plant-pathogen interaction | ko04626 | 405(2.09%) |
| Citrate cycle (TCA cycle) | ko00020 | 391(2.02%) |
| Cysteine and methionine metabolism | ko00270 | 386(1.99%) |
| Protein processing in endoplasmic reticulum | ko04141 | 377(1.94%) |
| Sulfur metabolism | ko00920 | 366(1.89%) |
| Peroxisome | ko04146 | 358(1.85%) |
| Spliceosome | ko03040 | 352(1.82%) |
| Fatty acid degradation | ko00071 | 346(1.78%) |
| Carbon fixation in photosynthetic organisms | ko00710 | 325(1.68%) |
| Amino sugar and nucleotide sugar metabolism | ko00520 | 321(1.66%) |
| Propanoate metabolism | ko00640 | 320(1.65%) |
| Phenylpropanoid biosynthesis | ko00940 | 315(1.62%) |
| Glutathione metabolism | ko00480 | 294(1.52%) |
| Plant hormone signal transduction | ko04075 | 287(1.48%) |
| RNA transport | ko03013 | 287(1.48%) |
| Fatty acid biosynthesis | ko00061 | 271(1.40%) |
| Butanoate metabolism | ko00650 | 264(1.36%) |
| RNA degradation | ko03018 | 263(1.36%) |
| beta-Alanine metabolism | ko00410 | 242(1.25%) |
| Biosynthesis of unsaturated fatty acids | ko01040 | 239(1.23%) |
| Pentose phosphate pathway | ko00030 | 237(1.22%) |
| Phenylalanine, tyrosine and tryptophan biosynthesis | ko00400 | 233(1.20%) |
| Endocytosis | ko04144 | 227(1.17%) |
| Tryptophan metabolism | ko00380 | 227(1.17%) |
| Carotenoid biosynthesis | ko00906 | 224(1.16%) |
| Pentose and glucuronate interconversions | ko00040 | 223(1.15%) |
| Porphyrin and chlorophyll metabolism | ko00860 | 221(1.14%) |
| Arginine and proline metabolism | ko00330 | 220(1.13%) |
| Valine, leucine and isoleucine biosynthesis | ko00290 | 220(1.13%) |
| Selenocompound metabolism | ko00450 | 220(1.13%) |
| alpha-Linolenic acid metabolism | ko00592 | 210(1.08%) |
| Arginine biosynthesis | ko00220 | 209(1.08%) |
| Terpenoid backbone biosynthesis | ko00900 | 203(1.05%) |
| Pantothenate and CoA biosynthesis | ko00770 | 202(1.04%) |
| Lysine degradation | ko00310 | 200(1.03%) |
| Biotin metabolism | ko00780 | 200(1.03%) |

# Supplementary Table S5. First 20 term of Go enrichment of R0vsR10 differentially expressed genes.

| GO Term (level 1) | GO Term (level 2) | | | Qvalue | | Gene number | | |
| --- | --- | --- | --- | --- | --- | --- | --- | --- |
|  |  |  |  |  |  | Up | Down | |
| Biological Process | protein phosphorylation | | | 3.1152E-55 | | 118 | 233 | |
|  | cellular protein modification process | | | 1.5107E-36 | | 210 | 325 | |
|  | protein modification process | | | 1.5107E-36 | | 210 | 325 | |
|  | cellular protein metabolic process | | | 5.8023E-15 | | 318 | 438 | |
|  | macromolecule modification | | | 3.4317E-13 | | 227 | 346 | |
|  | plant-type cell wall organization | | | 3.4317E-13 | | 2 | 21 | |
|  | plant-type cell wall organization or biogenesis | | | 3.4317E-13 | | 2 | 21 | |
| Molecular Function | | protein kinase activity | 1.157E-21 | | 127 | | | 246 |
|  |  | heme binding | 9.9025E-20 | | 68 | | | 108 |
|  |  | tetrapyrrole binding | 8.7897E-17 | | 70 | | | 110 |
|  |  | protein binding | 8.0009E-16 | | 494 | | | 603 |
|  |  | iron ion binding | 2.673E-14 | | 62 | | | 87 |
|  |  | phosphotransferase activity, alcohol group as acceptor | 1.2458E-13 | | 148 | | | 285 |
|  |  | metal ion binding | 3.7274E-13 | | 341 | | | 407 |
|  |  | calcium ion binding | 1.1205E-12 | | 44 | | | 55 |
| Cellular Component | | cell wall | 1.502E-18 | | 16 | | | 46 |
|  |  | nuclear ubiquitin ligase complex | 8.2463E-14 | | 50 | | | 28 |
|  |  | anaphase-promoting complex | 8.2463E-14 | | 50 | | | 28 |
|  |  | cullin-RING ubiquitin ligase complex | 8.2463E-14 | | 50 | | | 28 |
|  |  | ubiquitin ligase complex | 3.0544E-13 | | 50 | | | 28 |

# Supplementary Table S6. First 20 term of Go enrichment of R0vsR20 differentially expressed genes.

| GO Term (level 1) | GO Term (level 2) | Qvalue | Gene number | |
| --- | --- | --- | --- | --- |
|  |  |  | Up | Down |
| Biological Process | protein phosphorylation | 2.27E-20 | 106 | 102 |
|  | cellular protein modification process | 2.52E-13 | 174 | 158 |
|  | protein modification process | 2.52E-13 | 174 | 158 |
|  | defense response | 5.38E-11 | 31 | 65 |
|  | response to biotic stimulus | 3.65E-08 | 18 | 55 |
|  | plant-type cell wall organization | 5.92E-08 | 2 | 14 |
|  | plant-type cell wall organization or biogenesis | 5.92E-08 | 2 | 14 |
|  | cell wall organization | 6.04E-08 | 14 | 20 |
| Molecular Function | heme binding | 2.27E-20 | 62 | 81 |
|  | tetrapyrrole binding | 3.09E-18 | 64 | 83 |
|  | iron ion binding | 1.15E-17 | 57 | 70 |
|  | oxidoreductase activity, acting on paired donors, with incorporation or reduction of molecular oxygen | 1.25E-11 | 63 | 76 |
|  | microtubule motor activity | 5.67E-08 | 28 | 0 |
|  | protein binding | 5.67E-08 | 458 | 291 |
|  | microtubule binding | 1.05E-07 | 38 | 3 |
|  | transition metal ion binding | 1.05E-07 | 196 | 159 |
| Cellular Component | cell wall | 4.12E-14 | 16 | 31 |
|  | photosystem | 2.67E-08 | 47 | 11 |
|  | thylakoid | 1.45E-07 | 54 | 14 |
|  | thylakoid part | 1.45E-07 | 54 | 14 |

# Supplementary Table S7. Top 20 term of Go enrichment of R0vsR30 differentially expressed genes.

| GO Term (level 1) | GO Term (level 2) | Qvalue | Gene number | |
| --- | --- | --- | --- | --- |
|  |  |  | Up | Down |
| Biological Process | protein phosphorylation | 6.87E-49 | 161 | 170 |
|  | cellular protein modification process | 1.23E-26 | 251 | 236 |
|  | protein modification process | 1.23E-26 | 251 | 236 |
|  | pollination | 6.43E-12 | 8 | 26 |
|  | pollen-pistil interaction | 6.43E-12 | 8 | 26 |
|  | recognition of pollen | 6.43E-12 | 8 | 26 |
|  | cell wall organization | 1.70E-11 | 22 | 24 |
|  | multi-multicellular organism process | 2.52E-11 | 13 | 26 |
|  | cell recognition | 5.61E-11 | 8 | 26 |
|  | plant-type cell wall organization | 3.01E-10 | 5 | 15 |
|  | plant-type cell wall organization or biogenesis | 3.01E-10 | 5 | 15 |
| Molecular Function | protein kinase activity | 8.45E-19 | 175 | 178 |
|  | protein binding | 3.44E-15 | 630 | 428 |
|  | heme binding | 1.79E-14 | 75 | 82 |
|  | tetrapyrrole binding | 6.43E-12 | 78 | 82 |
|  | iron ion binding | 2.52E-11 | 63 | 73 |
|  | transferase activity, transferring hexosyl groups | 1.95E-10 | 53 | 69 |
|  | phosphotransferase activity, alcohol group as acceptor | 6.30E-10 | 203 | 197 |
|  | hydrolase activity, hydrolyzing O-glycosyl compounds | 8.49E-10 | 92 | 60 |
| Cellular Component | cell wall | 3.98E-15 | 24 | 32 |

# Supplementary Table S8. Top 20 term of Go enrichment of R20vsR30 differentially expressed genes.

| GO Term (level 1) | GO Term (level 2) | Qvalue | Gene number | |
| --- | --- | --- | --- | --- |
|  |  |  | Up | Down |
| Biological Process | protein phosphorylation | 3.47E-13 | 19 | 53 |
|  | cellular protein modification process | 4.08E-09 | 33 | 69 |
|  | protein modification process | 4.08E-09 | 33 | 69 |
|  | macromolecule modification | 5.81E-05 | 36 | 75 |
|  | phosphorylation | 1.54E-03 | 22 | 63 |
|  | protein ubiquitination | 3.00E-03 | 10 | 12 |
|  | protein modification by small protein conjugation | 3.28E-03 | 10 | 12 |
|  | protein modification by small protein conjugation or removal | 4.05E-03 | 11 | 13 |
| Molecular Function | protein kinase activity | 1.36E-07 | 20 | 58 |
|  | phosphotransferase activity, alcohol group as acceptor | 4.51E-05 | 25 | 62 |
| Molecular Function | xyloglucan: xyloglucosyl transferase activity | 1.00E-04 | 6 | 1 |
|  | kinase activity | 2.23E-04 | 26 | 64 |
|  | metal ion binding | 3.71E-04 | 55 | 81 |
|  | cation binding | 5.60E-04 | 56 | 82 |
|  | hydrolase activity, hydrolyzing O-glycosyl compounds | 1.54E-03 | 19 | 15 |
|  | heme binding | 3.00E-03 | 22 | 9 |
|  | hydrolase activity, acting on glycosyl bonds | 4.34E-03 | 20 | 15 |
|  | ubiquitin-protein transferase activity | 4.61E-03 | 9 | 12 |
|  | ubiquitin-like protein transferase activity | 4.61E-03 | 9 | 12 |
| Cellular Component | apoplast | 1.00E-04 | 6 | 1 |
